# Tailored Management of Anomalous Systemic Arterial Supply to the Basal Segment of the Lung: A Case Report and Literature Review

**DOI:** 10.70352/scrj.cr.26-0288

**Published:** 2026-05-23

**Authors:** Shunsuke Mori, Ken Suzawa, Koji Tomita, Haruchika Yamamoto, Kumi Nakajima, Shin Tanaka, Hidejiro Torigoe, Kazuhiko Shien, Kentaroh Miyoshi, Mikio Okazaki, Seiichiro Sugimoto, Takao Hiraki, Shinichi Toyooka

**Affiliations:** 1 Department of General Thoracic Surgery and Breast and Endocrinological Surgery, Okayama University Graduate School of Medicine, Dentistry and Pharmaceutical Sciences, Okayama, Okayama, Japan; 2 Department of Radiology, Okayama University Graduate School of Medicine, Dentistry and Pharmaceutical Sciences, Okayama, Okayama, Japan

**Keywords:** anomalous systemic arterial supply, basal lung segment, segmentectomy, endovascular embolization, pulmonary infarction

## Abstract

**INTRODUCTION:**

Parenchyma-preserving strategies for anomalous systemic arterial supply to the basal segment of the lung have gained increasing attention. However, pulmonary infarction of the preserved lung has been reported, and clear criteria for selecting the optimal treatment have yet to be established. We report 2 cases in which detailed preoperative imaging informed tailored management—right posterior basal segmentectomy in 1 patient and endovascular embolization of the aberrant artery in the other—both without postoperative complications. A review of the relevant literature is also provided, with an emphasis on potential selection criteria.

**CASE PRESENTATION:**

**Case 1:** A 20-year-old asymptomatic woman was referred after an abnormal screening chest radiograph. CT demonstrated an aberrant artery arising from the abdominal aorta supplying the right posterior basal segment (S10) with a large intravascular thrombus. The pulmonary artery showed hypoplasia limited to A10, while the other branches were normal, and no parenchymal congestion was identified. Following resection of the aberrant artery, robot-assisted right S10 segmentectomy was performed. The postoperative course was uneventful, and the patient was discharged on POD 6. **Case 2:** A 27-year-old woman was incidentally diagnosed on CT for an unrelated indication. An aberrant artery arising from the descending thoracic aorta supplied the left basal segment. Pulmonary arterial branches were preserved, with only minimal congestion in S9-10. Angiography revealed no evidence of an arteriovenous fistula. As surgical lung resection was considered unnecessary, coil embolization of the aberrant artery was performed. No complications occurred, and the patient was discharged on day 3 after the procedure.

**CONCLUSIONS:**

In patients with anomalous systemic arterial supply to the basal segment of the lung, when pulmonary arterial branches are preserved and background parenchymal congestion is minimal, parenchyma-sparing approaches should be considered.

## INTRODUCTION

Anomalous systemic arterial supply to the basal segment of the lung is a relatively rare congenital anomaly characterized by the presence of an aberrant artery arising from the aorta that supplies the basal segment despite normal bronchial and alveolar structures. This condition was previously classified as Pryce type I pulmonary sequestration^1)^; however, it is now regarded as a distinct disease entity.^2)^ Clinically, it carries several potential risks, including pulmonary hypertension due to left-to-left shunting, progressive pulmonary parenchymal damage, and aneurysmal dilatation or rupture of the aberrant artery; therefore, early intervention is generally recommended.^3)^ Although lobectomy was traditionally the standard treatment, advances in diagnostic imaging, surgical techniques, and endovascular therapy have broadened therapeutic options.^4)^ In addition to parenchyma-sparing approaches such as segmentectomy with resection of the aberrant artery, minimally invasive strategies—most notably coil embolization of the aberrant artery without pulmonary resection—have also emerged as viable alternatives.^5–7)^ However, clear criteria for selecting the optimal treatment strategy have not yet been established.

In this report, we describe 2 cases without clear pulmonary arterial absence and with no or only minimal congestion of the background lung parenchyma: one treated with segmentectomy and the other managed with coil embolization. We further discuss tailored management strategies based on each patient’s clinical and radiologic characteristics.

## CASE PRESENTATION

### Case 1

**Fig. 1:**
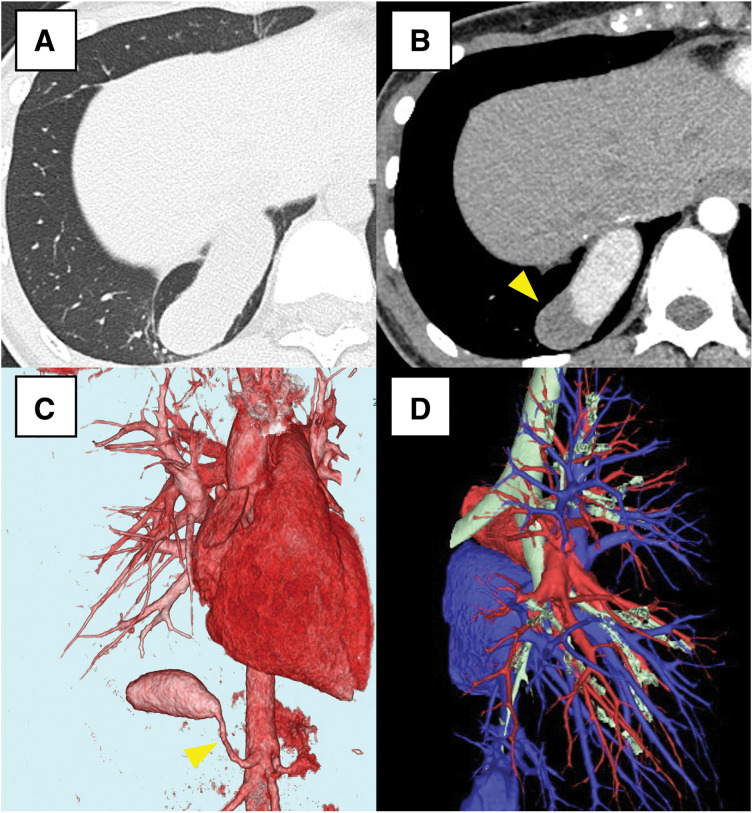
Preoperative contrast-enhanced chest CT scan of Case 1. (**A**) Lung window shows no congestive changes in the background lung parenchyma. (**B**) The mediastinal window shows an aberrant artery supplying the right S10, with intraluminal thrombus formation (yellow arrowhead). (**C**, **D**) 3D-CT reconstructions show an aberrant artery (yellow arrowhead) originating from the abdominal aorta. Hypoplasia is limited to A10, while the other pulmonary arterial branches are preserved.

A 20-year-old woman was referred to our institution after an abnormal shadow was detected in the right lower lung field on a chest radiograph obtained during a routine health checkup. Contrast-enhanced CT revealed an aberrant artery arising from the abdominal aorta at the T12 level, coursing dorsally into the thoracic cavity and supplying the right posterior basal segment (S10). The aberrant artery was slender within the abdominal cavity but became markedly dilated within the thoracic cavity, with a maximum diameter of approximately 18 mm, and intravascular thrombus formation was observed. The pulmonary arterial branches except A10 were preserved, and no congestive changes were observed in the background lung parenchyma. Although A10 was hypoplastic, no abnormalities of the pulmonary veins were identified (**Fig. 1**). Given the marked aneurysmal dilatation with thrombus limited to S10, resection of S10 together with division of the aberrant artery was considered necessary, whereas preservation of the other segments was deemed feasible. Accordingly, a right S10 segmentectomy was planned.

**Fig. 2:**
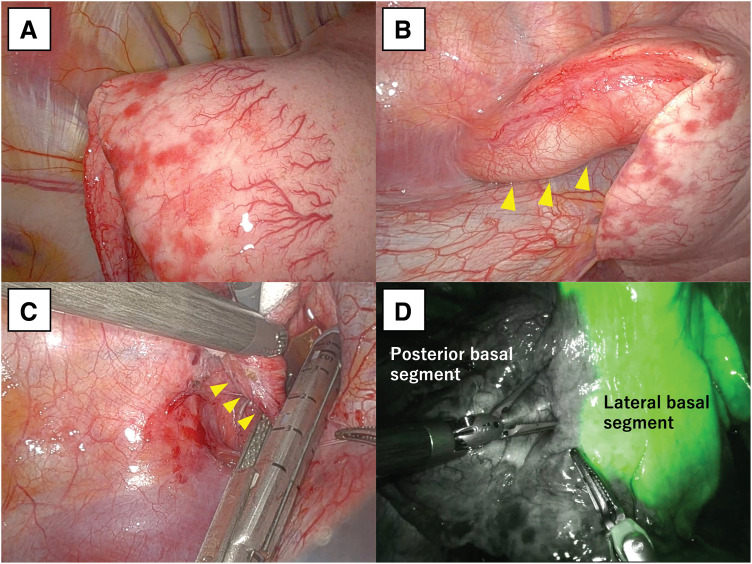
Intraoperative findings. (**A**) Dilated microvessels on the visceral pleura confined to S10. (**B**) A markedly dilated aberrant artery (yellow arrowheads) entering the thoracic cavity and supplying S10. (**C**) The central side of the aberrant artery (yellow arrowheads) was ligated with 1-0 silk suture, followed by division of the distal (pulmonary) side using a stapler. (**D**) The intersegmental planes delineating the posterior basal segment (S10) were clearly visualized after intravenous administration of indocyanine green.

Robot-assisted right S10 segmentectomy was performed. A markedly dilated aberrant artery was identified entering the thoracic cavity from the ventral side adjacent to the vertebral body and supplying S10. Dilated microvessels were observed on the visceral pleura, confined to S10. The aberrant artery was carefully dissected and exposed proximally toward the abdominal side until a narrowed segment was identified. At this narrowed portion, the artery was ligated with 1-0 silk suture, and the distal (pulmonary) side was divided using a hand-held stapler (Endo GIA stapler; Covidien, Mansfield, MA, USA). V10, B10, and the A10 branch were then divided sequentially. After intravenous administration of indocyanine green to delineate the intersegmental plane, the parenchyma was divided with staplers and S10 was resected (**Fig. 2**). The operative time was 164 min, and the estimated blood loss was 5 mL. The chest drain was removed on POD 2, and the patient was discharged on POD 6 without any complications.

Histopathological examination revealed a dilated elastic artery with intraluminal thrombus extending into the lung parenchyma, while no pathological abnormalities were observed in the background lung tissue. Follow-up CT performed 3 months after surgery showed no findings suggestive of pulmonary infarction, and the postoperative course has been uneventful.

### Case 2

A 27-year-old woman was referred to our institution after a mass-like lesion was detected at the left hilar region on a non-contrast chest CT performed for evaluation of right shoulder pain.

**Fig. 3:**
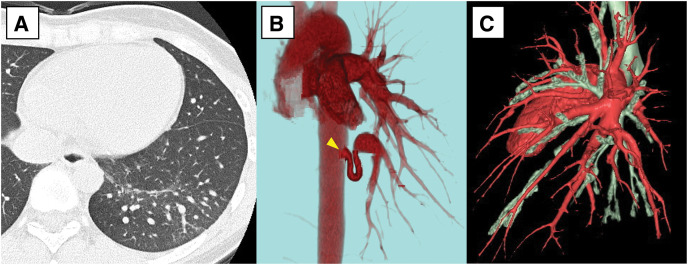
Preoperative contrast-enhanced chest CT scan of Case 2. (**A**) Lung window image showing extremely mild background pulmonary congestion localized to S9–10. (**B**, **C**) 3D-CT reconstructions showing an aberrant artery arising from the descending thoracic aorta (yellow arrowhead), with no pulmonary arterial defect.

Contrast-enhanced CT revealed an aberrant artery arising from the descending thoracic aorta at the T8 level and supplying the left basal segment. The maximum diameter of the aberrant artery was 9.5 mm. Pulmonary arterial branches were preserved, and congestion of the background lung parenchyma was minimal and limited to left S9-10. No abnormalities of the pulmonary veins were identified. Angiography showed that the aberrant artery bifurcated into 2 branches shortly after its origin and descended to supply the basal segment. No arteriovenous fistula was observed (**Fig. 3**). A multidisciplinary conference involving thoracic surgeons, pulmonologists, and radiologists concluded that pulmonary resection was unnecessary. Therefore, embolization of the aberrant artery was planned.

**Fig. 4:**
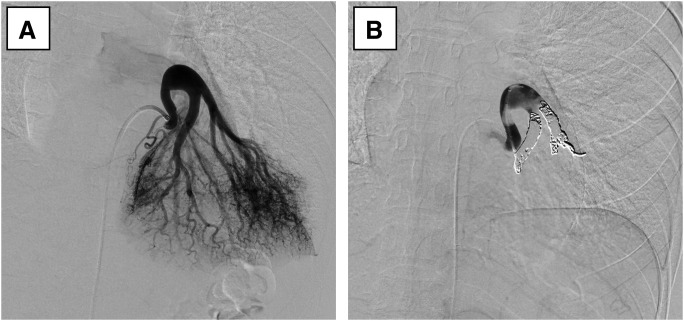
Angiography and coil embolization for the anomalous systemic artery. (**A**) DSA showing the aberrant artery from the thoracic aorta, bifurcating into 2 branches near its origin. (**B**) After coil embolization, interruption of blood flow in the anomalous artery was confirmed.

Coil embolization was performed for both the lateral and caudal branches of the aberrant artery, and complete interruption of blood flow was confirmed at the end of the procedure (**Fig. 4**). The post-procedural course was uneventful, and the patient was discharged on day 3 after the procedure. Contrast-enhanced CT at 3 months showed no findings suggestive of pulmonary infarction or pneumonia, and the clinical course was favorable.

### Literature review

A literature search was conducted in PubMed with the term “anomalous systemic arterial supply.” English-language case reports with full-text availability involving patients aged 15 years or older were included. A total of 48 case reports were identified. After excluding cases managed with lobectomy or conservative observation, 25 cases treated with segmentectomy, partial resection, aberrant artery division, or embolization were eligible for review.^8–25)^ Including the present 2 cases, 27 cases are summarized in **Table 1**.

**Table 1:** Anomalous systemic arterial supply to the basal lung segment: review of English literature

Age (years)	32 (15–78)
Sex, male/female	17 (63.0)/10 (37.0)
Symptoms (overlapping)	
Hemoptysis	11
Bloody sputum	3
Dyspnea on exertion	2
Others	2
Asymptomatic	12
Side, left/right/bilateral	21 (77.8)/5 (18.5)/1 (3.7)
Origin of aberrant arteries	
Descending thoracic aorta/celiac artery/	24/3/1
Abdominal aorta	
Diameter of aberrant artery (mm)^a^	12 (5–50)
Perfusion territory of the aberrant artery	
Left basal segment	18
Left S9–10	3
Left basal and lingular segment	1
Right S10	5
Right basal segment	1
Treatment	
Embolization	11
Segmentectomy	7
Wedge resection	5
Arterial division	5
Complications^b^	
None	19
(Embolization)	
Pulmonary infarction	1
Post-embolization syndrome	2
Recurrent hemoptysis	1
(Arterial division)	
Pneumonia	1

Reported English-language cases excluding lobectomy and conservative management, including the present cases. Data are n (%) or median (range).

^a^Missing data for 12 cases.

^b^Missing data for 3 cases.

Of these cases, 17 patients were male and 10 were female, with a mean age of 32.0 years. Approximately half of the patients presented with hemoptysis or blood-tinged sputum, while 12 patients were asymptomatic. Left-sided involvement was predominant, and 1 case of bilateral anomalous systemic arterial supply was reported.

Regarding the origin of the aberrant artery, the descending thoracic aorta was the most frequent source (24 cases; left:right = 21:3), followed by the celiac artery (3 cases; left:right = 1:2) and the abdominal aorta (1 case; right-sided). On the left side, aberrant arterial supply from the descending thoracic aorta to the basal segments was most common, whereas on the right side, aberrant arterial supply to the S10 region was most frequently observed.

With respect to treatment, embolization alone was performed in 11 cases, segmentectomy in 7 cases, division of the aberrant artery in 5 cases, and partial resection in 5 cases. Among patients treated with embolization, complications including pulmonary infarction or post-embolization syndrome occurred in 3 cases, and recurrent hemoptysis due to distal migration of the embolic device was observed in 1 case. One patient who underwent division of the aberrant artery developed early postoperative pneumonia, whereas no complications were reported in the remaining 19 cases. Importantly, in all 3 cases in which pulmonary infarction or post-embolization syndrome occurred after embolization, preoperative imaging demonstrated absence or hypoplasia of the pulmonary artery to the affected region. Furthermore, imaging findings suggestive of background parenchymal congestion were described in 2 of these cases.

## DISCUSSION

The present cases illustrate the feasibility of parenchyma-sparing management for anomalous systemic arterial supply to the basal segment of the lung when preoperative imaging suggests preserved native pulmonary arterial perfusion and limited background parenchymal changes. In both patients, detailed imaging evaluation supported a tailored strategy—segmentectomy in one patient and endovascular embolization in the other—without posttreatment complications.

A major concern with lung-preserving approaches is the risk of ischemic injury to the retained parenchyma after interruption of aberrant systemic flow. As summarized in **Table 1**, pulmonary infarction or post-embolization syndrome following embolization was reported exclusively in cases in which preoperative imaging demonstrated absence or hypoplasia of the pulmonary artery to the affected region. In addition, background parenchymal congestion was described in a subset of complicated cases. Although the number of reported events is small, these observations support the concept that the safety of parenchyma-sparing strategies depends on whether the target region is adequately perfused by the native pulmonary circulation once systemic inflow is eliminated.

Accordingly, a thorough preoperative imaging evaluation is essential when considering limited resection or embolization. Advances in imaging modalities, including 3D-CT in addition to conventional angiography, have enabled more precise and less invasive evaluation.^7)^ In particular, treatment selection should take into account 2 imaging factors: (i) the presence and severity of background parenchymal abnormalities suggestive of congestion, such as ground-glass opacity, consolidation, and interlobular septal thickening, and (ii) hypoplasia or absence of the pulmonary artery to the involved segment. Recent reports suggest that these parenchymal abnormalities are heterogeneous. Ground-glass opacity accompanied by consolidation may correspond pathologically to irreversible vascular degeneration and alveolar hemorrhage. These findings suggest that structural damage has already developed in some patients.^26)^ By contrast, interlobular septal thickening and increased parenchymal density may improve after interruption of aberrant systemic arterial flow, suggesting that at least part of the congestion-related changes can be reversible.^27)^ Therefore, when preoperative CT shows no conspicuous findings suggestive of moderate-to-severe background parenchymal congestion, particularly no ground-glass opacity with consolidation, and when native pulmonary arterial perfusion to the preserved lung is maintained, parenchyma-sparing approaches may be reasonable options. Conversely, in patients with insufficient pulmonary arterial supply to the affected region or with marked background parenchymal abnormalities, simple interruption of systemic inflow may predispose to infarction, persistent hemorrhagic damage, or infection of the preserved lung, and resection-based strategies may warrant stronger consideration.

With respect to our cases, Case 1 showed hypoplasia limited to A10, preservation of the other pulmonary arterial branches, and no evidence of background parenchymal congestion on contrast-enhanced CT. Therefore, an S10 segmentectomy was performed to remove the aneurysmally dilated aberrant artery with associated thrombosis, while preserving segments S7–9. Case 2 demonstrated minimal background parenchymal congestion and preserved pulmonary arterial branches on contrast-enhanced CT, and no arteriovenous fistula was identified on angiography. Given these findings, embolization was selected to preserve the lung parenchyma. Although favorable short- to mid-term outcomes after embolization have been reported, evidence regarding the long-term durability of embolization alone remains limited, including the risk of late recanalization.^9,28)^ For example, Jiang et al. reported complete occlusion of the anomalous artery in all 13 patients treated with transarterial embolization, with a mean CT follow-up of 25.4 months and a mean clinical follow-up of 42.1 months.^28)^ Nevertheless, long-term outcomes beyond these time frames have rarely been described. Therefore, when embolization alone is selected, particularly in young patients, as in Case 2, careful long-term imaging follow-up is necessary. In both cases, appropriate patient selection based on detailed preoperative imaging likely contributed to the absence of posttreatment complications.

This report has limitations inherent to a small number of cases and reliance on heterogeneous case reports in the literature. In particular, long-term outcomes after embolization alone remain insufficiently defined. Further accumulation of cases will be required to establish more definitive criteria for treatment selection.

## CONCLUSIONS

In cases of anomalous systemic arterial supply to the basal segment of the lung, accurate preoperative imaging assessment of background parenchymal congestion and the presence or absence of pulmonary arterial defects is essential for treatment selection. When pulmonary perfusion is preserved and parenchymal changes are minimal, parenchyma-sparing strategies should be considered.

## DECLARATIONS

### Availability of data and materials

The datasets supporting the conclusions of this article are included within the article and its figures; no additional data are available.
